# Physical Activity Is Associated With Lower Long-Term Incidence of Anxiety in a Population-Based, Large-Scale Study

**DOI:** 10.3389/fpsyt.2021.714014

**Published:** 2021-09-10

**Authors:** Martina Svensson, Lena Brundin, Sophie Erhardt, Ulf Hållmarker, Stefan James, Tomas Deierborg

**Affiliations:** ^1^Experimental Neuroinflammation Laboratory, Department of Experimental Medical Science, Lund University, Lund, Sweden; ^2^Center for Neurodegenerative Sciences, Van Andel Research Institute, Grand Rapids, MN, United States; ^3^Department of Physiology and Pharmacology, Karolinska Institute, Stockholm, Sweden; ^4^Department of Medical Sciences, Cardiology, Uppsala University, Uppsala, Sweden; ^5^Department of Internal Medicine, Mora Hospital, Mora, Sweden

**Keywords:** exercise, psychiatric disorders, mental health, women, men, long-term effect

## Abstract

Physical activity may prevent anxiety, but the importance of exercise intensity, sex-specific mechanisms, and duration of the effects remains largely unknown. We used an observational study design to follow 395,369 individuals for up to 21 years to investigate if participation in an ultralong-distance cross-country ski race (Vasaloppet, up to 90 km) was associated with a lower risk of developing anxiety. Skiers in the race and matched non-skiers from the general population were studied after participation in the race using the Swedish population and patient registries. Skiers (*n* = 197,685, median age 36 years, 38% women) had a significantly lower risk of developing anxiety during the follow-up compared to non-skiers (adjusted hazard ratio, HR 0.42). However, among women, higher physical performance (measured as the finishing time to complete the race, a proxy for higher exercise dose) was associated with an increased risk of anxiety compared to slower skiing women (HR 2.00). For men, the finishing time of the race did not significantly impact the risk of anxiety. Our results support the recommendations of engaging in physical activity to decrease the risk of anxiety in both men and women. The impact of physical performance level on the risk of anxiety requires further investigations among women.

## Introduction

Anxiety disorders are common mental health problems, estimated to currently affect up to 10% of the population globally (1) and twice as common among women compared to men (2, 3). The onset is typically early in life, during childhood, adolescence, or early adulthood. Additionally, co-morbidity with depression or other mental illnesses is common (2). Several reports reveal poorer physical health and shorter life expectancy among patients with anxiety disorders (4, 5). Unfortunately, up to half of the patients do not receive enough symptom relief when treated with first-line treatments, such as selective serotonin reuptake inhibitors (SSRI) or cognitive-behavioral therapy (CBT) (6). Due to the high prevalence, early-onset, and frequency of treatment-resistance among individuals with anxiety disorders, their contribution to years lived with disability and economic burden for society is substantial (7).

Physical activity has been pointed out as a promising strategy to mitigate the burden of anxiety, both when it comes to preventing the disease (8–10) as well as to alleviate the symptoms (11–13). Moreover, increasing the physical activity levels may also improve the physical health among these individuals, thereby reducing their comorbidity with other disorders and increasing their life expectancy.

Little is known when it comes to the impact of exercise dose, intensity or physical fitness level on the risk of developing anxiety disorders (14–17). Further, it remains unclear whether physical activity and fitness impact the risk of developing anxiety disorders equally in men and women. Importantly, several studies indicate that physical activity may affect anxiety levels differently among men and women (3, 10, 18–22).

Contrariwise, there are also studies indicating that physical activity may not reduce anxiety symptoms (23, 24), or at least not as much as psychopharmaceuticals do (23, 25). Further, it has been argued that the association between high physical activity and a lower risk of getting diagnosed with anxiety disorders may be driven by reverse causation (26–29). For instance, anxious symptoms before diagnosis may prevent vulnerable individuals from engaging in physical activity. Indeed, patients diagnosed with anxiety are less active than healthy controls (29–31) and stress has been indicated as a major obstacle for exercise participation according to a meta-analysis including psychiatric patients (32). As most studies in the field are cross-sectional, it is difficult to draw any conclusions regarding the causality in the association between physical activity and anxiety (30, 33–35). A recent meta-analysis including 14 prospective studies revealed that low physical activity predicted future anxiety (9). However, the longest follow-up time was <10 years and a majority of the included studies had <5 years of follow-up, which implicate a significant risk of bias due to reverse causation. Further, in a Swedish national cohort, Hallgren et al. followed over 27 000 middle-aged participants for 13 years (12). In their cross-sectional analysis, higher physical activity was associated with lower odds of anxiety symptoms, but no significant prospective association between activity and subsequent anxiety diagnosis was found. Long follow-up periods are needed and exclusion of individuals diagnosed with mental disorders within the first years after study inclusion to reduce the potential bias due to reverse causation.

We aimed to investigate the association between a physically active lifestyle and future development of anxiety disorders in men and women separately using a population-based cohort with a long-term perspective. Additionally, we investigate the impact of fitness level as a proxy for exercise dose on anxiety. We compared participants in the world's largest long-distance cross-country ski race (Vasaloppet) with matched non-skiers from the general population, to include a total of 395,369 individuals with up to 21 years of follow-up. To the best of our knowledge, the association between a physically active lifestyle and the development of anxiety disorders has not been investigated in such a large study population, including both men and women, with a long follow-up time before.

## Methods

### Study Design

This observational study design has been described previously (36) and has been approved by the Ethical Review Board in Uppsala, Sweden, (D.nr 2010/305). The study population includes all Swedes who participated in the world's largest long-distance (30–90 km), cross-country ski race (Vasaloppet) between 1989 and 2010 (*n* = 197,685), together with frequency-matched, individuals from the general population (*n* = 197,684) (Supplementary Figure 1). Statistics Sweden was used for frequency matching by drawing non-skier controls from the population registry according to region of residency, age group (5-year intervals), sex, and year of participation in ski race as described previously (37). In general, Vasaloppet skiers have higher leisure-time physical activity, smoke less, have a healthier diet, and lower mortality compared to the general Swedish population (38, 39). Individuals with severe disease were excluded as previously described (e.g., cancer, chronic neurologic disease, dementia, heart-, and lung disease) (40) to reduce bias due to the inability to participate in the race because of poor physical health. In addition, we excluded skiers and non-skiers with dementia [all-cause, Alzheimer's disease (AD), vascular dementia (VaD), Parkinson disease dementia, Lewy body dementia, senile dementia], Parkinson disease, meningitis/encephalitis, epilepsy, psychiatric disorders (depressive episode, schizophrenia, bipolar disorder, anxiety disorders, and mental disorders due to the use of alcohol) (see Supplementary Table 1).

In addition to ski race participation, skiers were monitored for finishing time in the race in three categories with finishing time of 100–150, 150–200, and above 200% of the winning finishing time for each sex, respectively. The finishing time analysis was used as a measurement of physical fitness and a proxy for the more extreme doses of exercise. Information on date of birth, sex, and education level was derived from Swedish registries (Swedish National Patient Registry for diagnoses and Statistics Sweden for socio-economic data) (37). The total study cohort (*n* = 395,369) was followed in the Swedish National Patient Registry (described below) throughout 2010.

### The Swedish National Patient Registry

The Swedish National Patient Registry was used to retrieve psychiatric and somatic diagnoses. It provides information on all primary and secondary diagnoses in patients attending hospital-based care in Sweden since 1987. The register additionally includes hospital-based out-patient visits since 2001 and covers 99% of all hospital-based diagnoses. Primary care diagnoses are not included in the registry. Anxiety disorders were defined according to the International Classification of Diseases (ICD), tenth revision (ICD10), or ninth revision (ICD9). Diagnoses included are (F40, F41, F42, 300A, 300B, 300C, 300D, 300D, 3000, 3001, 3002, 3003).

### Statistical Analyses

R statistical software package was used for analyses. *P* < 0.05 were considered statistically significant. Demographic data are presented as median and interquartile range (IQR) or numbers (n) and percent (%). Mann-Whitney *U* tests were used to estimate numeric group differences and categorical group differences were estimated with Pearson's χ^2^ test. Cox regression models were used to compare the risk of anxiety for skiers vs. non-skiers. The risks of anxiety disorders are presented as hazard ratios (HR) with 95% confidence intervals (CI). Numbers at risk were derived from survival tables specifying the number of individuals entering each 5-year interval, as presented in the graph. The time variable was calculated as years between participation in the ski race (and the same year for the matched non-skier) and event or censoring. The event was an anxiety disorder. Censoring appeared when subjects died or at the time of register outtake. Date of death for deceased study individuals was available through the Causes of Death Register (CDR), held at the National Board of Health and Welfare. Schoenfeld residuals were modeled graphically to assess the proportionality assumption. Men and women were also analyzed separately since sex was suggested to be a possible effect modifier. The impact of finishing time (fitness level) was assessed by trichotomizing the finishing time to 100–150%, 150–00%, and above 200% of the winning finishing time for men and women separately. Adjustments were done for sex, age, and education in the adjusted cox model. In primary sensitivity analyses, all individuals who developed anxiety disorders within 5 years of inclusion were excluded. In additional sensitivity analysis, all individuals who developed any psychiatric disorders (depression, anxiety, schizophrenia, or bipolar disorder, see Supplementary Table 1) within 5 years of inclusion were excluded.

## Results

### Ski Race Participation Is Associated With Lower Incidence of Anxiety

**Figure 1:**
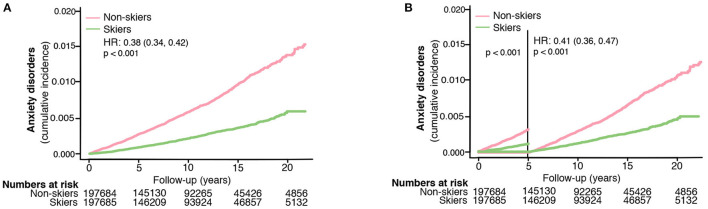
The risk of developing anxiety disorders in skiers compared to non-skiers **(A)** and the risk of developing anxiety disorders more than 5 years after completing the ski race **(B)**. HR represents hazard ratios from an unadjusted cox regression.

Table 1 shows the demographic data comparing the skiers and non-skiers. A total of 395,369 individuals were followed over 3975,881 person-years. After a median follow-up of 10 (IQR 5–15) years, a total of 1,649 individuals were newly diagnosed with anxiety disorders. Participation in the long-distance ski race was associated with a lower risk of developing anxiety disorders in the follow-up compared to non-skiers (unadjusted HR 0.38, 95% CI 0.34–0.42, Table 2, Figure 1A). Compared to non-skiers, skiers had a higher education than non-skiers (Table 1), but adjustments for age, sex, and education did not alter the results (adjusted cox model, Table 2). The effect remained even when individuals that developed anxiety within 5 years of the ski race (baseline) were excluded (unadjusted HR 0.41, 95% CI 0.36–0.47, Table 2, Figure 1B). Additional sensitivity analysis excluding all individuals who developed any psychiatric disorders within 5 years of inclusion did not alter the results (see Supplementary Table 2). Taken together, skiers in this race had a 62% lower relative risk of getting diagnosed with anxiety disorders compared to matched non-skiers.

**Table 1:** Characteristics of the study population, presented for the whole cohort, and by skiers and non-skiers separately.

	**All** ***n*** **= 395,369**	**Skiers** ***n*** **= 197,685**	**Non-skiers** ***n*** **= 197,684**
**Characteristics 1989–2010**	**Median (IQR) or** ***n*** **(%)**	**Median (IQR) or** ***n*** **(%)**	**Median (IQR) or** ***n*** **(%)**
Age at baseline, y	36.0 (29.0–46.0)	36.0 (29.0–46.0)	36.0 (29.0–46.0)
Women	149,796 (38)	74,897 (38)	74,899 (38)
**Education**			
Primary/elementary school (≤ 8 y)	49,344 (13)	14,538 (7.4)	34,806 (18)^***^
Secondary school/high school (9–12 y)	17,6571 (45)	76,635 (39)	99,936 (51)
Higher education/university (≥13 y)	16,6133 (42)	10,6147 (54)	59,986 (31)
**Anxiety disorders at follow-up**	1,649	456	1,193^***^

*IQR, interquartile range, y, years, n, numbers*.

*** *p < 0.001*.

*Group difference between skiers and non-skiers, estimated with Wilcoxon test (numeric variables) and Pearson's χ^2^ test (categorical variables)*.

*Only significant differences are noted in the table*.

**Table 2:** Association between physical activity and incident anxiety disorders, based on participation in a long-distance ski race (skiers) compared to non-skiers.

**Anxiety disorders**	**Unadjusted model**	Adjusted model^*^
**Physical activity**	**HR (95% CI)**	**HR (95% CI)**
***Nr events***	1,649	1,626
Non-skiers (Reference)	1	1
Skiers	0.38 (0.34–0.42)	0.42 (0.37–0.47)
**Excluding anxiety diagnoses** ** <5 years**		
***Nr events***	1,045	1,026
Non-skiers (Reference)	1	1
Skiers	0.41 (0.36–0.47)	0.44 (0.39–0.51)

*HR, hazard ratio; CI, confidence interval*.

*Cox regression models showing HR for risk of anxiety disorders*.

* *Model adjusted for age, sex, and education*.

### Both Male and Female Skiers Have Lower Incidence of Anxiety

**Figure 2:**
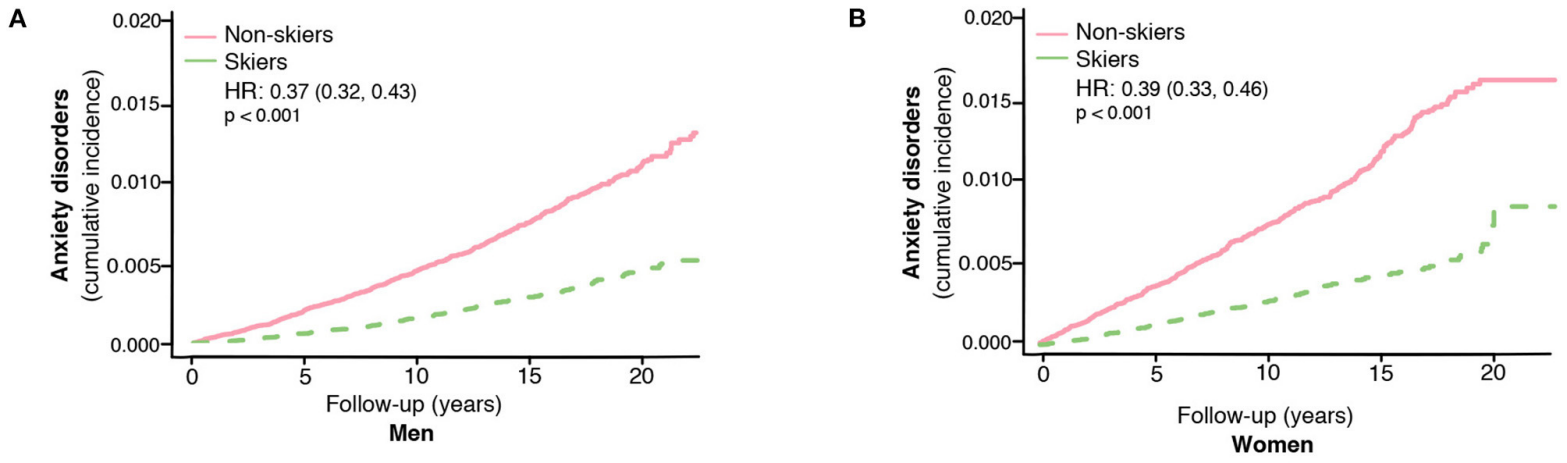
The risk of developing anxiety disorders in skiers compared to non-skiers in men **(A)** and women separately **(B)**. HR represents hazard ratios from an unadjusted cox regression.

The association between ski race participation and lower incidence of anxiety was seen in both men and women (unadjusted HR 0.37, 95% CI, 0.32–0.43 for men and unadjusted HR 0.39, 95% CI, 0.33–0.46 for women, Figures 2A,B).

### The Impact of Exercise Dose on Incident Anxiety Is Sex-Specific

**Figure 3:**
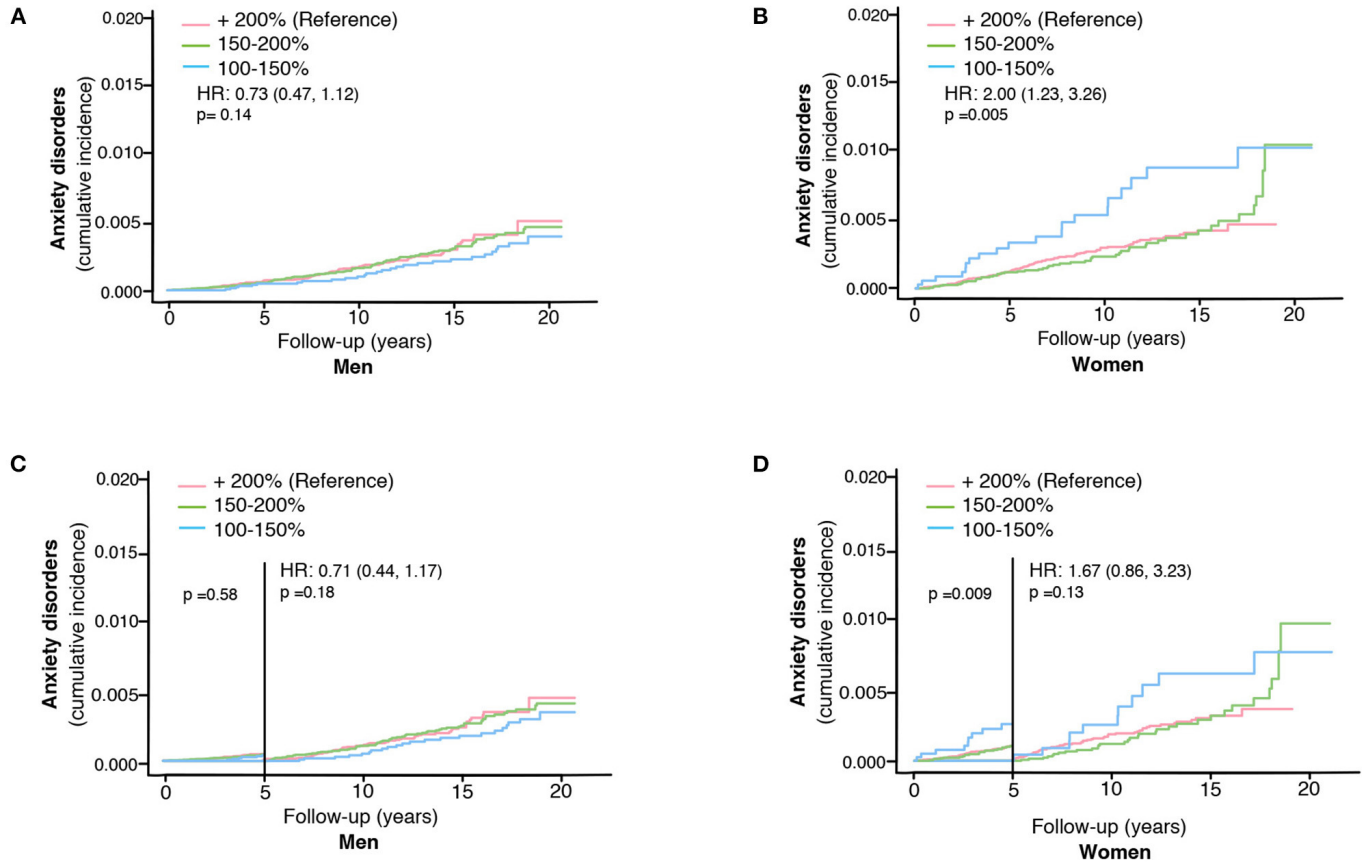
The impact of ski race finishing time on the risk of developing anxiety disorders in skiers in men **(A)** and women separately **(B)**. The impact of ski race finishing time on the risk of developing anxiety disorders more than 5 years after completing the ski race in men **(C)** and women **(D)**. HR represents hazard ratios from an unadjusted cox regression for the fastest (100–150% of winning finishing time) group, using +200% as the reference group.

We could not detect any impact of the ski race finishing time (a proxy for the effect of extreme exercise) on the risk of anxiety disorders among skiing men (unadjusted HR 0.73, 95% CI, 0.47–1.12, Table 3, Figure 3A). Opposingly, women completing the race with the shortest finishing time had a higher risk of developing anxiety compared to slower skiers (unadjusted HR 2.00, 95% CI, 1.23–3.26, Table 3, Figure 3B). Adjustments for age and education did not alter the results (adjusted cox model, Table 3). However, this association among the women became non-significant when excluding cases diagnosed with anxiety within the first 5 years (unadjusted HR 1.67, 95% CI, 0.86–3.23 for women, Table 3, Figures 3C,D).

**Table 3:** Association between ski race finishing time and incident anxiety disorders in men and women.

**Anxiety disorders**	**Men**	**Women**
**Finishing time (% of winning time)**	HR (95% CI)	HR (95% CI)
**Unadjusted model**		
> 200% (Reference)	1	1
150–200%	0.98 (0.73, 1.31)	0.99 (0.71, 1.38)
100–150%	0.73 (0.47, 1.12)	2.00 (1.23, 3.26)
Adjusted model^*^		
> 200% (Reference)	1	1
150–200%	0.95 (0.71, 1.27)	0.93 (0.66, 1.30)
100–150%	0.72 (0.46, 1.10)	1.72 (1.05, 2.83)
**Excluding anxiety diagnoses <5 years**		
**Unadjusted model**		
> 200% (Reference)	1	1
150–200%	1.00 (0.71, 1.41)	0.98 (0.63, 1.52)
100–150%	0.71 (0.44, 1.17)	1.67 (0.86, 3.23)
Adjusted model^*^		
> 200% (Reference)	1	1
150–200%	0.96 (0.68, 1.36)	0.92 (0.59, 1.43)
100–150%	0.71 (0.44, 1.17)	1.45 (0.74, 2.84)

*HR, hazard ratio; CI, confidence interval*.

*Cox regression models showing HR for risk of anxiety disorders in men and women, respectively*.

* *Model adjusted for age and education*.

## Discussion

We found that having a physically active lifestyle (being a skier) is associated with around 60% lower risk of developing anxiety disorders compared to matched individuals from the general population in an observational study following almost 400,000 individuals for up to 21 years. Our results were the same when excluding all individuals diagnosed with anxiety disorders within the first 5 years after study inclusion. Moreover, analysis of ski race finishing time (a proxy for the level of fitness) revealed a sex-specific association between the dose of exercise and incident anxiety.

Importantly, our study offers new knowledge about how a physically active lifestyle may affect the development of anxiety disorders in both men and women, adding to the findings made by Nyberg et al. They found low cardiovascular fitness to be associated with a higher risk of getting diagnosed with anxiety disorders in their study with up to 42-year follow-up of over 1 million Swedish men (8). As their study does not include women and as physical activity has been suggested to affect the risk of anxiety differently in men and women, our study adds important knowledge. We found participation in the ski race to be associated with a long-term lower risk of developing anxiety disorders in both men and women. This association remained when cases diagnosed within the first 5 years following inclusion were excluded.

We are not able to investigate the mechanisms behind the potential protective effects of exercise on the development of anxiety in our study. Nevertheless, several studies have tried to elucidate this. The ability of physical activity to pre-occupy the mind and offer distraction from other, potentially anxious, thoughts may explain its beneficial effects (41). As such, the natural environment during cross-country skiing may be specifically beneficial (42). Interestingly, physical activity has been shown to shift the recruitment of neurons in the rodent striatum during aversive events from those expressing dopamine D2 receptors, involved in stress vulnerability, toward others expressing D1 receptors involved in reward and stress resilience (43). Further, many patients with anxiety disorders have abnormal cortisol response after stress (44), and individuals with higher cardiovascular fitness or randomized to be physically active before being subjected to stress have a lower cortisol response (45). Moreover, exercise may reduce inflammation (46) and oxidative stress (47), systems suggested to be linked to anxiety (47–51), albeit investigated to a lesser extent compared to their involvement in depressive disorders (52–55). Exercise is also a well-known inducer of brain-derived neurotrophic growth factor (BDNF), which appears to be decreased in patients with anxiety disorders (56) and increasing levels have been linked to reduced anxiety in rodents following exercise (57, 58). However, the BDNF response to exercise seems to vary based on BDNF gene polymorphisms (59) and sex (58, 60, 61), where women tend to have less increase in BDNF following exercise (60, 61).

Interestingly, we found differences between men and women when analyzing the impact of finishing time of the ski race (a proxy for extreme exercise or higher fitness level) on the risk of anxiety disorders. Among male skiers, finishing time did not significantly affect the risk of developing anxiety disorders. However, among women, fast skiing was associated with a 2-fold higher risk of developing anxiety disorders compared to being a slower skier. Importantly, the cumulative incidence of anxiety disorders among fast skiing women was still lower than that of the matched non-skiing females from the general population. Thus, on a group level, physically high-performing women (fast skiers) may still benefit from a physically active lifestyle even though the optimal dose of exercise may be lower. To the best of our knowledge, this association between physical performance and the risk for anxiety disorders in women specifically has not been reported before. Previous studies have indicated that the effect of physical activity on anxiety may differ between men and women (3, 10, 18–22), but results are rather inconclusive. Some studies suggest that physical activity may have more pronounced effects against anxiety among women (21, 22, 62), whereas others report the opposite (10, 19). Interestingly, the impact of physical performance (being a fast skier) on the risk of anxiety disorders differs between male and female skiers in our study. Nyberg *et al*. reported that lower physical fitness was associated with a higher risk of developing anxiety disorders in their study, but that study only included males.

Even though our study does not investigate why faster skiing is associated with an increased risk of developing anxiety compared to slower skiers among women, possible reasons behind this has been discussed previously. For example, it may be caused by differences in the physiological response to exercise, where women have reported greater stress and exhaustion following exercise (19). Asztalos *et al*. suggested that the optimal intensity of physical activity to improve mental health may be high for men and milder for women (63). However, another study reveals a more beneficial effect of exercise on state anxiety in women if exercise was performed at a higher intensity (64). A possible explanation to the higher risk of anxiety among the fast skiing women in our study could be that confounding psychological factors linked to anxiety may be more frequent among these high-performing female skiers. For instance, appearance anxiety is more common among female exercisers (20, 65). Further, the individual's self-perception of physical fitness may correlate better with anxiety than the actual fitness level (66). These factors were not possible to investigate in our study, but female runners with pronounced physique anxiety are at higher risk for developing exercise dependence (67). Hence, psychological factors may drive a high exercise level in some of the high performing female skiers and this may be the reason behind their higher risk of anxiety. Thus, the relation between symptoms of anxiety and exercise behavior may not be linear. Even though many studies indicate that anxiety may prevent people from engaging in physical activity (30–32), it is also possible that symptoms linked to anxiety drive certain persons to exert more extreme exercise behaviors and this may be more pronounced among women (67). Consequently, the increased physical performance among these women may rather be a symptom of already present anxiety than causing anxiety disorders *per se*. Importantly, this association between faster skiing and higher risk for anxiety disorders among women becomes non-significant if individuals diagnosed during the first 5 years after inclusion are excluded. This indicates that this association may, at least to some extent, be driven by reverse causation. Studies investigating the driving factors behind these differences between men and women when it comes to extreme exercise behaviors are needed. In our recently published study on the development of depression in this study population, we saw a similar pattern regarding the difference in the impact of fast skiing on the risk for future depression among men and women (36). Future studies considering the impact of exercise intensity on the risk of developing anxiety disorders in men and women separately are warranted, especially with designs allowing for conclusions about directionality and causality of the association between physical activity and anxiety as our study design does not allow for these conclusions. An ongoing trial with exercise interventions of different intensities as a treatment for patients already diagnosed with anxiety will hopefully increase our knowledge regarding this within the near future (68).

Limitations of the study include that the physical activity level is not the only factor distinguishing our skiing population from their matched non-skiers in the general population. This population of skiers smokes less and has a better diet compared to the control population of non-skiers (38, 39). We were not able to control for this as we lack data on this for the majority of the participants. However, the results were not altered when we adjusted for age, sex, and education. Moreover, we do not have any detailed information about the physical activity in our cohort. We use baseline participation in the long-distance ski race (30–90 km) as a proxy of a physically active lifestyle. The race is physically demanding and requires preparatory exercise long term before the race. Nevertheless, it is possible that the reference group of non-skiers to some extent include physically active and this may attenuate the true association. Still, the participants in this ski race have reported a higher average time spent with physical activity than the matched non-skiing population (38, 39). Furthermore, as outcome measurement, we use anxiety diagnoses registered in the national wide patient registry. Although this registry is one of the largest in the world, and that diagnoses set in the primary care are likely to be imported into this registry given our long follow-up time, our data will only contain diagnoses and not the presence of anxiety symptoms. This means that our study does not consider the impact of symptoms related to undiagnosed anxiety disorders, which still may impact life quality and lifestyle physical activity. However, to reduce the influence of reverse causation on our results, we excluded individuals already diagnosed with severe disorders that may prevent their participation in the ski race. In our sensitivity analysis we additionally excluded those diagnosed with anxiety or other psychiatric disorders during the first 5 years after inclusion. Nonetheless, it is not possible to eliminate other factors that may lead to reverse causation, such as the influence of individual personality traits to exercise engagement and anxiety disorder vulnerability (11, 21, 26, 69). Therefore, we identify a need for future studies to gain deeper knowledge about the impact of these confounding psychological factors, taking both environmental, genetic, and epigenetic background into account.

In conclusion, our study setup offered a unique possibility to study the effect of a physically active lifestyle on the development of anxiety disorders by following 395,369 individuals during a period of up to 21 years and analyzing diagnoses set in the Swedish patient registry. We found that having a physically active lifestyle (being a skier) is associated with a substantially lower risk of developing anxiety disorders among both men and women.

To the best of our knowledge, this is the largest population-based study to date, confirming a long-term association of a physically active lifestyle on the later development of anxiety disorders in both men and women seen in previous studies with shorter follow up times. Our results suggest that the preventive effects of physical activity on anxiety disorders may be greater than previously reported. Randomized intervention trials, as well as long-term objective measurements of physical activity in prospective studies, are required to assess the validity and causality of this association.

## Data Availability Statement

The raw data supporting the conclusions of this article will be made available by the authors, without undue reservation.

## Ethics Statement

The studies involving human participants were reviewed and approved by Ethical Review Board in Uppsala, Sweden. Written informed consent for participation was not required for this study in accordance with the national legislation and the institutional requirements.

## Author Contributions

MS drafted the article, interpreted the results, and prepared the figures and tables. UH and SJ was responsible for setting up the Vasaloppet Registry. TD drafted the idea of our study. All authors participated in the discussion about how to analyse and interpret the results as well as critically revising the manuscript.

## Funding

We were funded by the Strategic Research Area MultiPark (Multidisciplinary Research in neurodegenerative diseases) at Lund University, the Swedish Alzheimer foundation, the Swedish Brain Foundation, Crafoord Foundation, Swedish Dementia Association, G&J Kock Foundation, Olle Engkvist Foundation, the Swedish Medical Research Council, the Swedish Parkinson Foundation, the A.E. Berger Foundation, the Thurings Foundation, and the Swedish mental health foundation. LB was supported by the National Institutes of Mental Health and the MJ Fox Foundation.

## Conflict of Interest

The authors declare that the research was conducted in the absence of any commercial or financial relationships that could be construed as a potential conflict of interest.

## Publisher's Note

All claims expressed in this article are solely those of the authors and do not necessarily represent those of their affiliated organizations, or those of the publisher, the editors and the reviewers. Any product that may be evaluated in this article, or claim that may be made by its manufacturer, is not guaranteed or endorsed by the publisher.
