# Supplementary material for: Physical Activity Is Associated With Lower Long-Term Incidence of Anxiety in a Population-Based, Large-Scale Study
Source: Front Psychiatry. 2021 Sep 10;12:714014. doi: 10.3389/fpsyt.2021.714014 (PMC8460768; doi:10.3389/fpsyt.2021.714014)
Supplement: Supplementary file 1 [file Data_Sheet_1.docx]

**Supplementary material**

**1. Detailed Method description**

**2. Supplementary Figure**

**3. Supplementary Tables**

**1. Detailed Method description**

**Matching procedure and exclusion criteria in the Vasaloppet study**

Since severe diseases are likely to hinder participation in a demanding long-distance race, individuals with such diseases were excluded. Supplementary Figure S1 shows a flow diagram describing numbers excluded due to severe disease. The ICD-codes of these diagnoses have been stated previously(1). In addition, we excluded participants with diagnoses presented in Table S1.

Moreover, during the initial matching process, a control individual from the general population was assigned for every ski race. Thus, skiers participating in Vasaloppet several times got several controls. To get equally many skiers as non-skiers, we therefore completed a re-matching procedure. However, as we only used the index race for each skier, the non-skiing group would have been older if we had included one control for every time a skier participated in the race.

**2. Supplementary Figure**

**Vasaloppet Study population**

Supplementary Figure S1. Flow diagram describing the Vasaloppet Study population.

**3. Supplementary Tables**

**Supplementary Table 1. Exclusion criteria (additional)**

| **Diagnosis** | **ICD-9** | **ICD-10** |
| --- | --- | --- |
| Anxiety disorders | 300A, 300B, 300C, 300D, 300D, 3000, 3001, 3002, 3003 | F40, F41, F42 |
| Bipolar disorder | 296A, 29610 ,296C, 296D, 296E, 29600, 29610, 29620, 29630, 29688, 29699 | F30, F29, F310, F311, F312, F313, F314 ,F315, F316, F317, F318, F319 |
| Depressive episode | F399, 296B, 296X, 29620, 29800 | F32, F33, F34, F38 |
| Schizophrenia | 295, 297, 2970, 2979, 29999 | F20, F21, F22, F23, F24, F25, F28, F29 |
| Mental disorders due to the use of alcohol | 291, 2910, 2919 | F10 |
| Alzheimer´s disease | 331A/3310, 29010 | F00, G30 |
| Vascular dementia | 290E, 2904, 2930 | F01 |
| All-cause dementia | 290, F070, 294C, 294B, 331A, 310A, G318A | F00, F01, F02, F03, G30 |
| Lewy body dementia | 331X, G318A, 33182 | F028 |
| Dementia in Parkinson disease | 294B, 332A | F023 |
| Parkinson disease | 332A, 3420 | G20 |
| Meningitis/encephalitis | 3200, 320A, 320B, 320C, 320D, 320W 320X, 321A, 321B, 321C, 321D, 321E, 321X, 322A, 322B, 322C, 320X, 323, 3230 | G00, G01, G03, G04, G05 |
| Epilepsy | 345, 3450 | G40 |

**Supplementary Table 2. Additional sensitivity analyses**

| **Anxiety disorders** | ***Unadjusted model*** | ***Adjusted model**** |
| --- | --- | --- |
| **Excluding psychiatric diagnoses** <5 years** |  |  |
| ***Nr events*** | 1007 | 990 |
| Non-skiers (Reference) | 1 | 1 |
| Skiers | 0.41 (0.36-0.47) | 0.45 (0.39-0.51) |

HR: hazard ratio, CI: confidence interval

Cox regression models showing HR for risk of anxiety disorders.

*Model adjusted for age, sex, and education.

** Excluding all individuals who developed any psychiatric disorders (depression, anxiety, schizophrenia, or bipolar disorder, see Supplementary Table 1) within five years of inclusion.

**References**

1. Hallmarker U, Michaelsson K, Arnlov J, Hellberg D, Lagerqvist B, Lindback J, et al. Risk of recurrent ischaemic events after myocardial infarction in long-distance ski race participants. Eur J Prev Cardiol. 2016;23(3):282-90.
